# EFS: an ensemble feature selection tool implemented as R-package and web-application

**DOI:** 10.1186/s13040-017-0142-8

**Published:** 2017-06-27

**Authors:** Ursula Neumann, Nikita Genze, Dominik Heider

**Affiliations:** 1 Straubing Center of Science, Schulgasse 22, Straubing, 94315 Germany; 2 University of Applied Science, Weihenstephan-Triesdorf, Freising, 85354 Germany; 3 Wissenschaftszentrum Weihenstephan, Technische Universität München, Freising, 85354 Germany

**Keywords:** Machine learning, Feature selection, Ensemble learning, R-package

## Abstract

**Background:**

Feature selection methods aim at identifying a subset of features that improve the prediction performance of subsequent classification models and thereby also simplify their interpretability. Preceding studies demonstrated that single feature selection methods can have specific biases, whereas an ensemble feature selection has the advantage to alleviate and compensate for these biases.

**Results:**

The software EFS (Ensemble Feature Selection) makes use of multiple feature selection methods and combines their normalized outputs to a quantitative ensemble importance. Currently, eight different feature selection methods have been integrated in EFS, which can be used separately or combined in an ensemble.

**Conclusion:**

EFS identifies relevant features while compensating specific biases of single methods due to an ensemble approach. Thereby, EFS can improve the prediction accuracy and interpretability in subsequent binary classification models.

**Availability:**

EFS can be downloaded as an R-package from CRAN or used via a web application at http://EFS.heiderlab.de.

## Background

In the field of data mining, feature selection (FS) has become a frequently applied preprocessing step for supervised learning algorithms, thus a great variety of FS techniques already exists. They are used for reducing the dimensionality of data by ranking features in order of their importance. These orders can then be used to eliminate those features that are less relevant to the problem at hand. This improves the overall performance of the model because it addresses the problem of overfitting. But there are several reasons that can cause instability and unreliability of the feature selection, e.g., the complexity of multiple relevant features, a small-n-large-p-problem, such as in high-dimensional data [1, 2], or when the algorithm simply ignores stability [3, 4]. In former studies, it has been demonstrated that a single optimal FS method cannot be obtained [5]. For example, the Gini-coefficient is widely used in predictive medicine [6, 7], but it has also been demonstrated to deliver unstable results in unbalanced datasets [8, 9]. To counteract instability and therewith unreliability of feature selection methods, we developed an FS procedure for binary classification, which can be used, e.g., for random clinical trials. Our new approach ensemble feature selection (EFS) [10] is based on the idea of ensemble learning [11, 12], and thus is based on the aggregation of multiple FS methods. Thereby a quantification of the importance scores of features can be obtained and the method-specific biases can be compensated. In the current paper we introduce an R-package and a web server based on the EFS method. The user of the R-package as well as the web application can decide which FS methods should be conducted. Therewith, the web server and the R-package can be applied to perform an ensemble of FS methods or to calculate an individual FS score.

## Implementation

We used existing implementations in **R** (http://www.r-project.org/) for our package EFS. The following section will briefly introduce our methodology. For deeper insights please refer to [10]. Our EFS currently incorporates eight feature selection methods for binary classifications, namely median, Pearson- and Spearman-correlation, logistic regression, and four variable importance measures embedded in two different implementations of the random forest algorithm, namely *cforest* [9] and *randomForest* [13].

### Median

This method compares the positive samples (class = 1) with negative samples (class = 0) by a Mann-Whitney-U Test. The resulting *p*-values are used as a measure of feature importance. Thus, a smaller *p*-value indicates a higher importance.

### Correlation

We used the idea of a fast correlation based filter of of Yu and Liu [14] to select features that are highly correlated with the dependent variable, but show only low correlation with other features. The fast correlation based filter eliminates features with high correlation with other features to avoid multicollinearity. The eliminated features get an importance value of zero. Two correlation coefficients, namely the Pearson product-moment and the Spearman rank correlation coefficient were adopted and their *p*-values were used as importance measure.

### Logistic regression

The weighting system (i.e., *β*-coefficients) of the logistic regression (LR) is another popular feature selection method. As preprocessing step a Z-transformation is conducted to ensure comparability between the different ranges of feature values. The *β*-coefficients of the resulting regression equation represent the importance measure.

### Random forest

Random forests (RFs) are ensembles of multiple decision trees, which gain their randomness from the randomly chosen starting feature for each tree. There are different implementations of the RF algorithm in R available, which offer diverse feature selection methods. On the one hand we incorporated the *randomForest* implementation based on the classification and regression tree (CART) algorithm by Breiman [13]. The *cforest* implementation from the party package, on the other hand, uses conditional trees for the purpose of classification and regression (cf. [15]). In both implementations an error-rate-based importance measure exists. The error-rate-based methods measure the difference before and after permuting the class variable. Due to their dependency on the underlying trees, results are varying for both error-rates. The *randomForest* approach also provides an importance measure based on the Gini-index, which measures the node impurity in the trees. Whereas in *cforest* an AUC-based variable importance measure is implemented. The AUC (area under the curve) is the integral of the receiver operating characteristics (ROC) curve. The AUC-based variable importance measure works to the error-rate-based one, but instead of computing the error rate for each tree before and after permuting a feature, the AUC is computed.

### Ensemble learning

The results of each individual FS methods are normalized to a common scale, an interval from 0 to $\frac {1}{n}$, where *n* is the number of conducted FS methods chosen by the user. Thereby we ensure the comparability of all FS methods and conserve the distances between the importance of one feature to another.

### R-package

The EFS package is included in the Comprehensive R Archive Network (CRAN) and can be directly downloaded and installed by using the following R command:

 In the following, we introduce EFS’s three functions ensemble_fs, barplot_fs and efs_eval. A summary of all commands and parameters is shown in Table 1.

**Table 1:** Method overview

Command	Parameters	Information
ensemble_fs	data	object of class data.frame
	classnumber	index of variable for binary classification
	NA_threshold	threshold for deletion of features with a greater proportion of NAs
	cor_threshold	correlation threshold within features
	runs	amount of runs for randomForest and cforest
	selection	selection of feature selection methods to be conducted
barplot_fs	name	character string giving the name of the file
	efs_table	table object of class matrix retrieved from ensemble_fs
efs_eval	data	object of class data.frame
	efs_table	table object of class matrix retrieved from ensemble_fs
	file_name	character string, name which is used for the two possible PDF files.
	classnumber	index of variable for binary classification
	NA_threshold	threshold for deletion of features with a greater proportion of NAs
	logreg	logical value indicating whether to conduct an evaluation via logistic regression or not
	permutation	logical value indicating whether to conduct a permutation of the class variable or not
	p_num	number of permutations; default set to a 100
	variances	logical value indicating whether to calculate the variances of importances retrieved
		from bootstrapping or not
	jaccard	logical value indicating whether to calculate the Jaccard-index or not
	bs_num	number of bootstrap permutations of the importances
	bs_percentage	proportion of randomly selected samples for bootstrapping

The R-package EFS provides three functions

### ensemble_fs

The main function is ensemble_fs. It computes all FS methods which are chosen via the selection parameter and gives back a table with all normalized FS scores in a range between 0 and $\frac {1}{n}$, where *n* is the number of incorporated feature selection methods. Irrelevant features (e.g., those with too many missing values) can be deleted.



The parameter data is an object of class data.frame. It consists of all features and the class variables as columns. The user has to set the parameter classnumber, which represents the column number of the class variable, i.e., the dependent variable for classification. NA_threshold represents a threshold concerning the allowed proportion of missing values (NAs) in a feature column. The default value is set to 0.2, meaning that features with more than 20% of NAs are neglected by the EFS algorithm. The cor_threshold parameter is only relevant for the correlation based filter methods. It determines the threshold of feature-to-feature correlations [14]. The default value of cor_threshold is 0.7. The results of RF-based FS methods vary due to the randomness of their underlying algorithms. To obtain reliable results, the RF methods are conducted several times and averaged over the number of runs. This parameter, namely runs, is set to 100 by default. The user can select the FS methods for the EFS approach by using the selection parameter. Due to the high computational costs of the RFs, the default selection is set to



meaning that the two FS methods of the conditional random forest are not used by default.

### barblot_fs

The barblot_fs function sums up all individual FS scores based on the output of ensemble_fs and visualizes them in an cumulative barplot.



The barplot_fs function uses the output of the ensemble_fs function, namely the efs_table, as input. The parameter name represents the filename of the resulting PDF, which is saved in the current working directory.

### efs_eval

The efs_eval function provides several tests to evaluate the performance and validity of the EFS method. The parameters data, efs_table, file_name, classnumber and NA_threshold are identical to the corresponding parameters in the ensemble_fs function: 

#### Performance evaluation by logistic regression

The performance of the EFS method can automatically be evaluated based on a logistic regression (LR) model, by setting the parameter logreg = TRUE. efs_eval uses an LR model of the selected features with a leave-one-out cross-validation (LOOCV) scheme, and additionally trains an LR model with all available feature in order to compare the two LR models based on their ROC curves and AUC values with ROCR [16] and pROC based on the method of DeLong et al. [17]. A PDF with the ROC curves is automatically saved in the working directory.

#### Permutation of class variable

In order to estimate the robustness of the resulting LR model, permutation tests [18, 19] can be automatically performed, by setting the parameter permutation = TRUE. The class variable is randomly permuted p_num times and logistic regression is conducted. The resulting AUC values are then compared with the AUC from the original LR model using a Student’s t-Test. By default, p_num is set to 100 permutations.

#### Variance of feature importances

If the parameter variances is TRUE an evaluation of the stability of feature importances will be conducted by a bootstrapping algorithm. The samples are permuted for bs_num times and a subset of the samples (bs_percentage) is chosen to calculate the resulting feature importances. By default, the function chooses 90% of the samples and uses 100 repetitions. Finally, the variances of the importance values are reported.

#### Jaccard-index

The Jaccard-index measures the similarity of the feature subsets selected by permuted EFS iterations:

$$ J\left(S_{1},\ldots,S_{n}\right)=\frac{|S_{1}\cap\ldots\cap S_{n}|}{|S_{1}\cup\ldots\cup S_{n}|}, $$

where *S*_*i*_ is the subset of features at the *i*-th iteration, for *i*=1,…,*n*. The value of the Jaccard-index varies from 0 to 1, where 1 implies absolute similarity of subsets. If jaccard = TRUE is set, the Jaccard-index of the subsets retrieved from the bootstrapping algorithm is calculated.

### Availability and requirements

The package is available for R-users under the following requirements:

- **Project name:** Ensemble Feature Selection
- **Project home page (CRAN):** http://cran.r-project.org/web/packages/EFS
- **Operating system (s):** Platform independent
- **Programming language:** R (≥ 3.0.2)
- **License:** GPL (≥ 2)
- **Any restrictions to use by non-academics:** none

Due to the high relevance of our EFS tool for researchers who are not very familiar with R (e.g., medical practitioners), we also provide a web application at http://EFS.heiderlab.de. It contains the functions ensemble_fs and barplot_fs. Therefore no background knowledge in R is necessary to use our new EFS software.

## Results

**Fig. 1:**
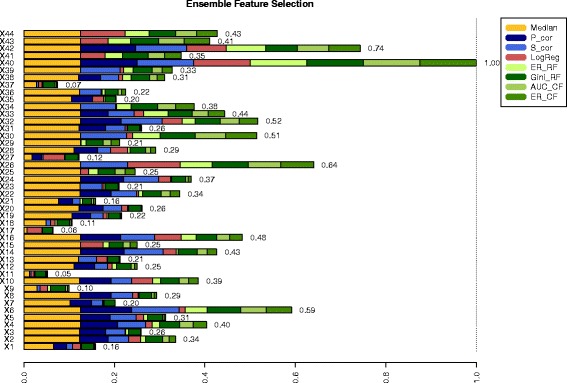
Cumulative barplot retrieved from barplot_fs function of R-package EFS



**Fig. 2:**
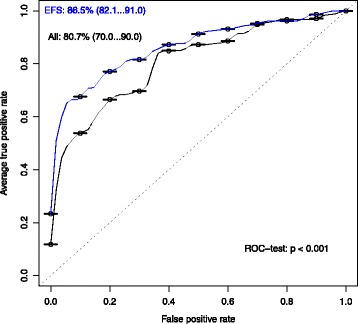
Performance of LR model. On the *y-axis* the average true positive rate (i.e., sensitivity) and on the *x-axis* the false positive rate (i.e., 1-specificity) is shown. Two ROC curves are shown: of all features (*black*) and the EFS selected features (*blue*). The *dotted line marks* the performance of random guessing

**Fig. 3:**
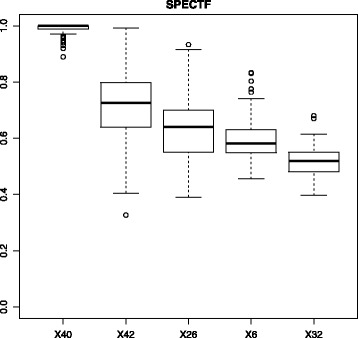
Boxplot of importances retrieved from the bootstrapping algorithm

The dataset SPECTF has been obtained from the UCI Machine Learning Repository [20] and is used as an example. It describes diagnosing of cardiac Single Proton Emission Computed Tomography (SPECT) images. The class-variable represents normal (= 0) and abnormal (= 1) results and can be found in the first column of the table of the file SPECTF.csv at the UCI repository. In general, the EFS approach accepts all types of data, i.e., all types of variables, except categorical variables. These variables have to be transformed to dummy variables in advance. Data has to be combined in a single file with one column indicating the class variable with 1 and 0, e.g., representing patients and control samples, or, e.g., positive and negative samples. After loading the dataset, we compute the EFS and store it in the variable “efs”:



The results can be visualized by the barplot_fs function:

 The output is a PDF named “SPECTF.pdf”. Figure 1 shows this cumulative barplot, where each FS method is given in a different color. Various methods to evaluate the stability and reliability of the EFS results are conducted by the following command:

The user retrieves two PDF files. Firstly, the resulting ROC curves of the LR test (“SPECTF_ROC.pdf”) including the p-value, according to Fig. 2. The p-value clearly shows that there is a significant improvement in terms of AUC of the LR with features selected by the EFS method compared the LR model without feature selection. Additionally, Fig. 3 shows the file “SPECTF_Variances.pdf”, in which boxplots of the importances retrieved from the bootstrapping approach are given. The calculated variances can be accessed in the eval_tests output. A low variance implies that the importance of a feature is stable and reliable.

An additional example is provided in the documentation of the R-package on a dataset consisting of weather data from the meteorological stations in Frankfurt(Oder), Germany in February 2016.

## Conclusion

The EFS R-package and the web-application are implementations of an ensemble feature selection method for binary classifications. We showed that this method can improve the prediction accuracy and simplifies the interpretability by feature reduction.

## Abbreviations

AUC: Area under the curve
CART: Classification and regression tree
CRAN: Comprehensive R archive network
EFS: Ensemble feature selection
FS: Feature selection
LR: logistic regression
LOOCV: leave-one-out cross validation
RF: random forest
ROC: receiver operating characteristic
